# Supplementary material for: A cost-effective breast cancer screening strategy for Urban China: Findings from a Shenzhen-based modeling study
Source: PLoS One. 2026 Mar 12;21(3):e0344253. doi: 10.1371/journal.pone.0344253 (PMC12981465; doi:10.1371/journal.pone.0344253)
Supplement: S1 Fig — (DOCX) [file pone.0344253.s007.docx]

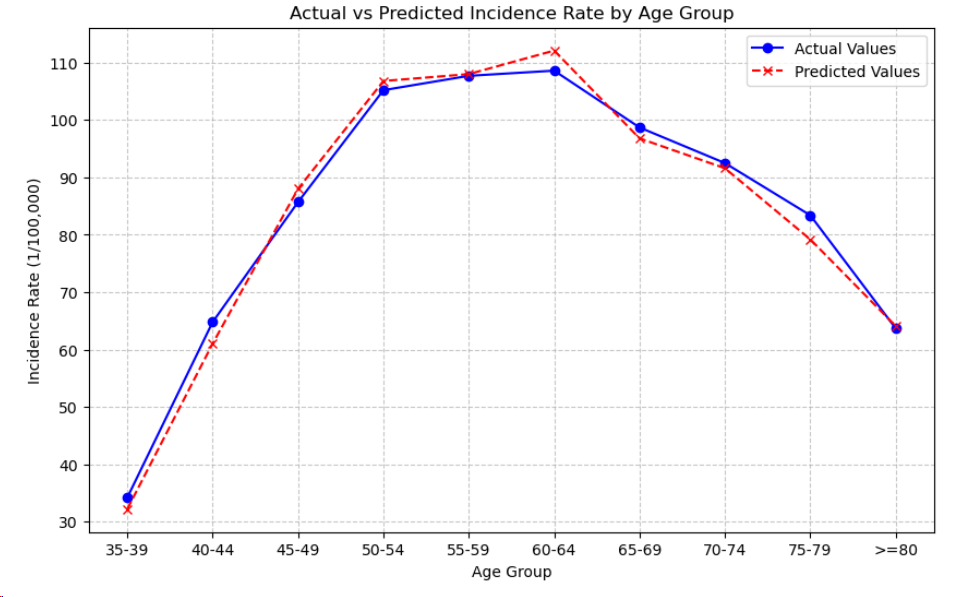


Supplementary figure 1 Comparison of predicted age-specific incidence rates and actual age-specific incidence rates curve of female breast cancer in ShenZhen.
