# Supplementary material for: A cost-effective breast cancer screening strategy for Urban China: Findings from a Shenzhen-based modeling study
Source: PLoS One. 2026 Mar 12;21(3):e0344253. doi: 10.1371/journal.pone.0344253 (PMC12981465; doi:10.1371/journal.pone.0344253)
Supplement: S1 Appendix — (DOCX) [file pone.0344253.s008.docx]

# ****Supplementary Appendix for**** ****"********A Cost-Effective Breast Cancer Screening Strategy for Urban China: Findings from a Shenzhen-Based Modeling Study"****

## A.1 Detailed Screening Protocol and Clinical Pathway

This study is a retrospective study based on data from the Breast Cancer Screening Project in Shenzhen. The data used in this study cover the period from 01/01/2021 to 31/12/2023. These data were accessed on 09/01/2024 for cost-effectiveness analysis. All data were fully anonymized before our analysis. We used these data to evaluate the current strategy of the screening program and to simulate various scenarios, including no screening, different starting and ending ages, and varying screening frequencies. The screening results were categorized using the Breast Imaging Reporting and Data System (BI-RADS) into several grades: BI-RADS I and II indicate negative results (no abnormalities); BI-RADS III indicates suspicious results, requiring further examination; BI-RADS IV and V indicate positive results, suggesting the uncertainty of breast cancer risk. BI-RADS 0 indicates incomplete or insufficient information, necessitating re-examination. Participants first underwent clinical breast examinations and ultrasound. If these tests yielded positive results (BI-RADS IV or V), a biopsy (either fine-needle aspiration or core needle biopsy) was performed for diagnosis. For those with suspicious or insufficient results (BI-RADS III or 0), mammography was conducted. Positive mammography results also necessitated a biopsy for confirmation, while suspicious or insufficient mammography results required clinical judgment for further action (see Figure 1 in the main text).

## ****A.2 Formal Markov Model Specification and State Transition Structure****

**A.2.1 Model Structure and State Definitions**

A state-transition Markov model was constructed using TreeAge 2011 software[1], to evaluate long-term breast cancer screening decisions. The model structure was adapted from an established Hong Kong breast cancer screening study [2], and is depicted in Figure 2.

The model's health state space $\boldsymbol{S}$ comprises seven mutually exclusive states:

$S$= {(Healthy (H),Ductal Carcinoma In Situ (D/DCIS),Stage I (S1),Stage II (S2),Stage III (S3),Stage IV (S4),Death (X) )}.

From the Healthy (H) state, an individual may remain healthy or transition to DCIS or any invasive cancer stage (I-IV). Within the clinical cancer states (DCIS, S1-S4), individuals may remain in their current stage, progress to a more advanced stage (e.g., S1→S2, S2→S4), or transition to the Death (X) state. Death (X) is modeled as an absorbing state in subsequent cycles, meaning no transitions out of this state are possible.

**A.2.2 State Transition Probability Matrix**

The model utilizes a non-homogeneous Markov transition matrix P(a), where the transition probabilities are functions of the cohort's attained age (a). , where each element $P_{ij}\left( a \right)$，denotes the probability of moving from state$i$ to state$j$. A value of 0 indicates a disallowed direct transition within a single cycle[2]. The matrix structure, which defines allowable transitions, is as follows:

$$P\left( a \right)= \left[ \begin{matrix} P_{HH}(a) & P_{HD}(a) & P_{HS1}(a) & P_{HS2}(a) & P_{HS3}(a) & P_{HS4}(a) & P_{Hx}(a) \\ 0 & P_{DD}(a) & P_{DS1}(a) & P_{DS2}(a) & P_{DS3}(a) & P_{DS4}(a) & P_{Dx}(a) \\ 0 & 0 & P_{S1S1}(a) & P_{S1S2} & 0 & P_{S1S4} & P_{S1x}(a) \\ 0 & 0 & 0 & P_{S2S2}(a) & P_{S2S3} & P_{S2S4} & P_{S2x}(a) \\ 0 & 0 & 0 & 0 & P_{S3S3}(a) & P_{S3S4} & P_{S3x}(a) \\ 0 & 0 & 0 & 0 & 0 & P_{S4S4}(a) & P_{S4x}(a) \\ 0 & 0 & 0 & 0 & 0 & 0 & 1 \end{matrix} \right]$$

Notation: D denotes DCIS (Ductal Carcinoma In Situ); H, Healthy; S1-S4, Stage I-IV; X, Death.

**Matrix Element Definitions and Sources:**

Time-homogeneous Elements: The annual probabilities of progression between invasive clinical stages are modeled as constants, independent of age. These values are sourced directly from Table 3 (eg., $P_{S1S2}$ (0.06), $P_{S2S3}$ (0.11), $P_{S1S4}$ (0.01), $P_{S2S4}$ (0.08), and $P_{S3S4}$ (0.21)).

Self-Transition Elements: The diagonal elements $P_{S1S1}(a)$, $P_{S2S2}(a)$, $P_{S3S3}(a)$, and $P_{S4S4}(a)$ represent the probability of remaining in the same disease state within a cycle. They are derived as the complement of the sum of outgoing transition probabilities from that state (e.g., $P_{S1S1}(a)$=1−$P_{S1S4}-P_{S1X}(a)$). Their values are therefore implicitly defined by the progression constants in Table 3 and the corresponding mortality rates.

Age-dependent Composite Elements: The transitions from the Healthy state to cancer states: The transitions from the Healthy state to cancer states-$P_{HD}(a)$, $P_{HS1}(a)$, $P_{HS2}(a)$, $P_{HS3}(a)$, $P_{HS4}(a), P_{DS1}(a),P_{DS2}(a),P_{DS3}(a),and P_{DS4}(a)$-are composite, age-dependent parameters derived through a two-step procedure. First, the age-specific all-cause mortality rate (Table 1) is applied to define the surviving cohort. Second, surviving individuals are distributed to cancer states based on age-specific incidence (Table 2) and the diagnostic pathway (from Table 8), which splits them into those detected through screening and those identified through the natural history path. Each group is then assigned to specific cancer states using the corresponding column in Table 4 (“Screening” or “No screening”).For example, $P_{HS2}(a)$ uses the Stage II proportion from Table 4: 0.3881 for screen-detected and 0.4570 for natural-history-path cases.

Mortality Transitions: $P_{iX}(a)$ elements represent the sum of stage-specific breast cancer mortality and age-specific non-breast cancer mortality derived from Table 1 and Table 3.

**A.2.3 Cohort Simulation Approach**

The study simulated a closed cohort of 100,000 women, all starting in the "Healthy" state at age 35. The model ran for 50 annual cycles, simulating the lifetime of the cohort until age 85. This time horizon was chosen to capture the significant increase in breast cancer incidence after age 35 and the majority of the population's lifespan.

In each annual cycle, individuals could transition between health states based on predefined probabilities. The cohort simulation method was used to calculate the distribution of individuals across all health states at the end of each cycle. The model tracked the accumulation of costs and Quality-Adjusted Life Years (QALYs) for each individual throughout their lifetime. Costs and QALYs were assigned based on the health state occupied in each cycle. The model output for each screening strategy was the average lifetime cost and average lifetime QALYs per woman.

## A.3 Model Calibration and Validation

To ensure the model's natural history module accurately reflects real-world disease progression in the absence of screening, we performed a rigorous model calibration process. The objective was to align the model-predicted age-specific breast cancer incidence rates with empirically observed rates from the target population [3].

The iterative calibration process was conducted as follows:

1. The model was run under a 'no-screening' scenario.

2. The model-generated age-specific incidence rates were systematically compared against the target empirical rates.

3. When discrepancies were identified, key unobservable natural history parameters—primarily the age-specific probabilities of symptomatic presentation—were systematically adjusted. For instance, if the model under-predicted incidence in later stages, the symptom detection probability for earlier stages was cautiously lowered, allowing a greater proportion of simulated cases to progress before clinical diagnosis.

4. This process was repeated until a pre-specified goodness-of-fit criterion was met. After 42 iterations, a satisfactory fit was achieved.

The goodness-of-fit between the final model output and the observed data was formally assessed using a Chi-square goodness-of-fit test. The test compared the model-predicted number of cases against the observed number of cases across 10 age strata (35-39, 40-44, ..., 75-80), resulting in 9 degrees of freedom. The test yielded a statistic of χ² = 2.366 with a p-value of 0.968. Since the p-value greatly exceeds the significance level (α = 0.05), we fail to reject the null hypothesis, indicating no statistically significant difference between the model-predicted and the observed incidence distributions (Supplementary Figure 1).

This successful calibration confirms that our model's "no-screening" baseline provides a scientifically robust and contextually valid foundation for comparing the incremental cost-effectiveness of the various screening strategies evaluated in this study.

## A.4 Cost-Utility Analysis

All screening strategies were ranked from lowest to highest based on total lifetime costs. An incremental analysis was then conducted to compare each more costly strategy with the next less costly, non-dominated alternative. The primary outcome of this analysis was the Incremental Cost-Utility Ratio (ICUR), defined as the additional cost per additional Quality-Adjusted Life Year (QALY) gained.

Within the TreeAge software, dominated strategies are categorized into Absolutely Dominated Strategies and Extended Dominated Strategies. The process for identifying these strategies is as follows:

1. Absolute Dominance: A strategy is considered absolutely dominated if it is both less effective (provides fewer QALYs) and more costly than another strategy.
2. Extended Dominance: A strategy is considered extendedly dominated if its ICUR is higher than that of a more effective strategy. This implies that a linear combination of two other strategies can achieve the same health benefit at a lower cost.

Dominated strategies were sequentially removed to identify the cost-effective frontier, which comprises only the Undominated Strategies. A full list and detailed description of all 27 screening strategies evaluated in this study are provided in Supplementary Table 2.

### **A.4.1 Calculation of Lifetime Costs and QALYs**

For each screening strategy $s$, the discounted lifetime cost ($C_{s}$) and QALYs ($Q_{s}$) per person are calculated as:

$$C_{s}=\sum_{t=1}^{T} \frac{c_{s}\left( t \right)}{\left( 1+r \right)^{t-1}},Q_{s}=\sum_{t=1}^{T} \frac{u_{s}\left( t \right)}{\left( 1+r \right)^{t-1}}$$

where:

- $c_{s}\left( t \right)$:total cost accrued in $cycle\left( t \right)$.
- $u_{s}\left( t \right)$: health utility weight (0-1) in $cycle(t)$
- $r$: annual discount rate 3%.
- $T$: time horizon (50 cycles).

### A.4.2 Incremental Analysis and Dominance Removal

Strategies were ranked by increasing cost $( C\_1 \leq C\_2 \leq\ldots\leq C\_n )$

1.Absolute Dominance:Strategy $k$ is absolutely dominated if $Q_{k}\leq Q_{j}$ and $C_{k}\geq C_{j}$for any other strategy $j$.

2. Incremental Cost-Utility Ratio (ICUR): The ICUR of strategy kk compared to the next non-dominated strategy $j$ is:

$$ICUR_{kJ}=\frac{C_{k}-C_{j}}{Q_{k}-Q_{j}}$$

3. **Extended Dominance:** Strategy $k$ is extendedly dominated if its ICUR is higher than that of a more effective strategy $\boldsymbol{m}$.
The process of identifying and removing dominated strategies iterates until only the cost-effective frontier remains.

## A.5 Sensitivity Analysis

This study employed sensitivity analysis to assess the robustness of the cost-effectiveness results to parameter uncertainty. We conducted both deterministic (one-way) and probabilistic analyses. In the deterministic analysis, key parameters (costs, sensitivity, specificity, health utilities) were varied across their plausible ranges, derived from our observed data and the literature, to identify influential inputs.

To evaluate the combined impact of all parameter uncertainties on the Incremental Cost-Utility Ratio (ICUR), we performed a probabilistic sensitivity analysis using Monte Carlo simulation with 1,000 iterations. In each iteration, parameter values were drawn simultaneously from predefined probability distributions: Gamma for costs, Beta for sensitivity and specificity, and Log-normal for health utility values, following established methodological guidelines[4, 5].

The distributions were parameterized so that their central tendencies reflected the base-case estimates. For costs, a constant coefficient of variation was assumed to shape the Gamma distributions. For all parameters, the specific distributional parameters (e.g., shape and rate for Gamma) were derived from the base-case values and their associated uncertainty ranges presented in the main text (Tables 5-8). The fully specified parameters for all probability distributions used in the Monte Carlo simulations are provided in Supplementary Tables 3-6, which correspond directly to the base-case inputs in main-text Tables 5-8, ensuring complete transparency and reproducibility of the probabilistic analysis.

1. Geisler BP. Automating first-and second-order Monte Carlo simulations for Markov models in TreeAge pro: INTECH Open Access Publisher; 2011.

2. Wong IO, Kuntz KM, Cowling BJ, Lam CL, Leung GM. Cost effectiveness of mammography screening for Chinese women. Cancer. 2007;110(4):885-95.

3. Wang J, Greuter MJW, Zheng S, van Veldhuizen DWA, Vermeulen KM, Wang Y, et al. Assessment of the Benefits and Cost-Effectiveness of Population-Based Breast Cancer Screening in Urban China: A Model-Based Analysis. Int J Health Policy Manage. 2022;11(9):1658-67.

4. Xiaomin W, Chongqing T, Xiaohui Z, luoxia, yilidan, Medicine CJoNDaC. Parameter distribution of associated parameters in probability sensitivity analysis of pharmacoeconomic evaluation. Chinese Journal of New Drugs and Clinical Medicine. 2018;37(3):3.

5. Briggs A. Probabilistic analysis of cost-effectiveness models: statistical representation of parameter uncertainty. Value in health : the journal of the International Society for Pharmacoeconomics and Outcomes Research. 2005;8(1):1-2.
