# Supplementary material for: A cost-effective breast cancer screening strategy for Urban China: Findings from a Shenzhen-based modeling study
Source: PLoS One. 2026 Mar 12;21(3):e0344253. doi: 10.1371/journal.pone.0344253 (PMC12981465; doi:10.1371/journal.pone.0344253)
Supplement: Attachment — Submitted filename: PONE-D-25-24977_Review.pdf [file pone.0344253.s009.pdf]

Review of "Cost-Effectiveness Analysis of Breast Cancer Screening for Women in Shenzhen, China: A Markov Modeling Study"

Summary

This manuscript presents a Cost-Effectiveness analysis and builds a Markov model to compare 27 possible breast cancer screening schedules for women in Shenzhen. Each schedule considers the combined Clinical Breast Examination (CBE), Breast Ultrasound (BUS), and supplementary Mammography (MAM), and mainly varies the screening frequency/interval and the start/stop ages. For every included schedule, the authors calculate incremental cost-utility ratios (ICURs) and run one-way sensitivity analyses to identify the most cost-effective schedule. While the study objective is interesting and important to the local society, I have reservations about the manuscript's suitability for the PLOS ONE journal. First, the manuscript needs thorough editing for language and presentation: it contains confusing descriptions, vague passages, inconsistent figure formatting, and incorrect in-text citation and reference-list styles. And because of these issues, it is difficult to read. Second, the novelty appears limited, as the methodological contribution seems limited because the modelling and analysis framework is similar to the referenced paper of Wong et al. (2007).

Comments

1. Pages 10 and 11. The literature evidence you described is not truly "contentious"; rather, each study answers a slightly different question. The literature is heterogeneous, but not contradictory. The summaries here are confusing, incomplete, and the writing flow between different studies needs to be improved. For instance, the text summary of the work from "Li et al. (2017)", the manuscript only states ICURs for breast ultrasound and mammography, but does not mention whether these modalities are cost-effective compared with no screening or not. (The related reference item is incomplete.) The same holds for several other papers cited in this paragraph. The words are confusing, incomplete, and the logic between different studies needs to be improved.

2. Figure 1. Since the focus of this paper is screening, the clinical assessment flow chart may not be related to this study.

3. Pages 13 and 14. The Markov model transition states are well defined. But the model descriptions are confusing; for example, the model input and outcomes are not clearly described. Also, you may want to expand with more details on the method for the cohort simulation using the fitted Markov model.

4. Page 16. "By iteratively adjusting the symptom probability in the model, we ensured that the distribution of cases presented at each stage aligned closely with the reported

distribution of incident cases." These words are confusing as well. What does it mean to iteratively adjust the symptom, and what are the iterations during the calculation?

5. Page 16. Although it is mentioned in the paper, "A goodness-of-fit test was conducted to evaluate the similarity between the two curves." But there are no hypothesis testing results or conclusions stated in the paper, i.e., reject or fail to reject the null hypothesis, test statistic, and p-value. The paper only mentioned, "This validation approach helps", but no words mentioning whether the validation is successful or not.

6. Page 17. The title of Table 3 is "Average annual progression probability of breast cancer staging", which needs to be adjusted since the table also includes other information beyond transition probabilities. Besides, "RR of invasive cancer from DICS" has a value of 2.02, which is, of course, not a probability, and there is no interpretation of the number, or an interpretation of what is RR's meaning is in the main text (Is it relative risk? If so, "RR" needs to be stated in full when you first time using it). The description here needs to be fine-tuned, and the table title needs to be adjusted.

7. Page 19. Table 5, the reference to "Shenzhen breast cancer screening program" should be corrected.

8. Page 26. Figure 3, this is the key graph in this paper, but it is very difficult to recognize the related screening strategy from the plot. Additionally, the phrases "upper-left region" and "lower-right corner" don't match what the figure actually shows—no points appear in either quadrant. A better summary and description of the plot is needed.

9. Pages 30 and 32. Figure 8 is displayed in the main text, but is not mentioned in any text. On page 30, you may want to describe what the color (red VS. green) means, one or two red dots are further apart from the confidence interval bound ellipse and the associated possible interpretation, etc.

10. Page 35. "with an ICUR of 140,915 CNY/QALY-well below China's societal WTP threshold of 537,000 CNY/QALY. " Based on your previous statement, 53,7000 CNY is calculated from the Shenzhen per capital GDP value, not the whole of Chinese societal WTP threshold.

11. The conclusions from comparisons between CBE+BUS_MAM/2year/35-65 with no screen and CBE+BUS_MAM/3year/45-65 with no screen are less interesting, especially in Shenzhen city with relatively higher GDP and hence higher threshold for cost-effectiveness.

12. Numerous citation and bibliography errors remain. Only two examples are given here,

   - In-text citations (page 15) – "Wong et al.'s research" lacks the publication year; the citations "(Wu et al., 2017)" and "(Y Huang et al., 2013)" are repeated in the same sentence and should appear only once.
   - Reference list – Several entries do not follow the required style:
     - Page 42, "wuli, chenguozhen, . . . " – authors' surnames need initial capitals ("Wuli, Chenguozhen, . . . ").
     - Some records omit journal titles or volume/issue details. i.e., "Zulfiqar, M., Rohazly, I., Rahmah, M. J. B. i., & journal, i. (2011). Do the majority of Malaysian women have dense breasts on mammogram? 7(2)."

References

Wong, I. O., Kuntz, K. M., Cowling, B. J., Lam, C. L., & Leung, G. M. (2007). Cost effectiveness of mammography screening for chinese women. *Cancer: Interdisciplinary International Journal of the American Cancer Society*, *110*(4), 885–895.
