# Supplementary material for: A cost-effective breast cancer screening strategy for Urban China: Findings from a Shenzhen-based modeling study
Source: PLoS One. 2026 Mar 12;21(3):e0344253. doi: 10.1371/journal.pone.0344253 (PMC12981465; doi:10.1371/journal.pone.0344253)
Supplement: Attachment — Submitted filename: PONE-D-25-24977_Review_R1.pdf [file pone.0344253.s011.pdf]

Review of "Cost-Effectiveness Analysis of Breast Cancer Screening for Women in Shenzhen, China: A Markov Modeling Study"

Summary

Thank you for the detailed, point-by-point response. The quality of the article has improved substantially. Shifting the tone from statistical analysis to study findings has strengthened the manuscript. However, I believe that the statistical analysis in Supplementary Appendix 1 still has considerable room for improvement and would benefit from more professional revision. Additionally, the in-text citations are still incorrect.

Comments

1. For A.2.2 State transition probability matrix, to have a more statistically professional sound, the description of the states could be like "From the Healthy states, an individual may remain Healthy, Ductal Carcinoma In Situ, Stage I, Stage II, ..., etc.". Also, for example, "Death in the last row of $P(t)$ is modelled as an absorbing state, with no transition out. ", etc.

2. For A.2.2 State transition probability matrix, the math notations were only used here. It would be better if the authors could link the non-zero matrix elements with the numbers in Table 3 in the main text so that the readers can easily connect them in math.

3. Is the transition dependent on age or time? Or is the model time-homogeneous within each age group? It means that for a given age stratum, the transition probabilities are constant over time. The matrix itself is labelled as $P(t)$, which suggests "transition matrix at time t", but the entries there are not functions of t in terms of the math expression. (It seems it is time homogeneous since there is only one group of state transition probabilities provided.) The description is very confusing.

4. For A.5 (also description in main text), "Each model parameter is assigned a specific probability distribution (Xiaomin et al., 2018): screening and treatment costs follow a Gamma distribution, sensitivity parameters follow a Beta distribution, and health utility value parameters follow a Log-normal distribution (Briggs, 2005). " I guess here you want to mention the model input parameters following either the Gamma distribution or Log-normal distribution, and these parameters were extracted from the above two literatures? I am a little bit confused since what I expect to read are the distribution parameters for $Gamma(\alpha, \beta)$, $Beta(a, b)$, and $Log-normal(\mu, \sigma^2)$ distributions when you talk about those distributions. Anyway, the distribution or model parameters should be clearly referenced between the description and the tables 6 and 8.

5. Some in-text citation still not correct, “(L. Sun et al., 2018; X. Zhang, 2015)”, “(L. Sun et al., 2018)”, “(I. O. Wong et al., 2007)”, “Wong et al(2007) (Wong et al., 2007)”;

6. Table cross-article reference: “(Wang et al., 2022;Table 2)”;

7. Figure references shouldn’t have a caption after the figure index, i.e. “Fig 2 simplified”;

8. Also, the *PLOS One* journal uses the Vancouver style? In-text citation should all be numbered citations like “[12]” in the text, with the full reference in the reference list? You shouldn’t use author-year at all.
