# Supplementary material for: A cost-effective breast cancer screening strategy for Urban China: Findings from a Shenzhen-based modeling study
Source: PLoS One. 2026 Mar 12;21(3):e0344253. doi: 10.1371/journal.pone.0344253 (PMC12981465; doi:10.1371/journal.pone.0344253)
Supplement: Attachment — Submitted filename: Response to Reviewer 1.docx [file pone.0344253.s012.docx]

**Response to Comments**

We extend our sincere gratitude to the reviewers for their time and valuable insights, which have significantly helped us improve the manuscript. Below, we provide a point-by-point response to the comments raised.

#### Response to Reviewer Comment #1

**Comment :** The literature evidence you described is not truly “contentious”; rather, each study answers a slightly different question. The literature is heterogeneous, but not contradictory. The summaries here are confusing, incomplete, and the writing flow between different studies needs to be improved. For instance, the text summary of the work from “Li et al. (2017)”, the manuscript only states ICURs for breast ultrasound and mammography, but does not mention whether these modalities are cost-effective compared with no screening or not. (The related reference item is incomplete.) The same holds for several other papers cited in this paragraph. The words are confusing, incomplete, and the logic between different studies needs to be improved.

**Response:**

We sincerely thank the reviewer for this critical and constructive observation. We completely agree that the term "contentious" was inappropriate and that our initial literature summary lacked clarity, completeness, and a logical narrative flow. The reviewer is correct in noting that the existing literature is best characterized by its heterogeneity in terms of study settings, populations, and compared strategies, rather than by direct contradiction.

In direct response to this comment, we have thoroughly revised the corresponding paragraph in the **Introduction**. Specifically, we have restructured the narrative to logically group studies based on their primary focus (e.g., single-modality vs. combined strategies, national vs. regional analyses) and to explicitly state the comparator and cost-effectiveness conclusions for each cited study. And we have Standardized the reference format throughout this section to ensure all citation items are complete and conform to the journal's style, addressing the issue of incomplete references.

#### Response to Reviewer Comment #2

We thank the reviewer for raising this thoughtful point regarding Figure 1. We agree that the primary focus of our paper is on the screening strategy and its cost-effectiveness. However, we included Figure 1 to precisely illustrate the full operational protocol of the screening program that we are evaluating, which includes the necessary clinical assessment steps for individuals with abnormal or inconclusive initial screening results.

We believe this figure is essential as it provides a clear and accurate definition of the (CBE+BUS)+MAM screening strategy evaluated in our model. The flowchart explicitly outlines the sequence of the three modalities—clinical breast examination, breast ultrasonography, and mammography—and specifies the clinical conditions under which mammography is utilized as a supplementary tool. This level of operational detail is crucial for ensuring that the model's structure faithfully reflects real-world clinical practice. Furthermore, the illustration connects the initial screening activities to their subsequent clinical outcomes by mapping the pathway from a positive or suspicious finding to a pathological diagnosis via biopsy. Capturing this full trajectory is fundamental to our cost-effectiveness analysis, as the resource use and clinical outcomes associated with these diagnostic steps are integral to the model's assessment of both costs and health benefits. Presenting the strategy without this critical pathway would offer an incomplete picture of the clinical and economic processes being evaluated.

To better align the figure with the paper's focus and to address the reviewer's valid concern, we have taken the following action in the revised manuscript:

We have revised the title of Figure 1 from "The Screening Flow Chart" to more accurately describe its content. The new title is: "Figure 1. Screening and Clinical Assessment Pathway for the（CBE+BUS)+MAM Strategy."

This change clarifies that the figure depicts not just the initial screening moment but the subsequent, essential clinical management pathway that is part and parcel of the overall screening program we evaluated.

We hope that with this clarification and the revised figure title, the relevance and necessity of Figure 1 are now clear. We are grateful for the reviewer's comment, which has helped us improve the clarity of our manuscript.

#### Response to Reviewer Comment #3

Comment:Pages 13 and 14. The Markov model transition states are well defined. But the model descriptions are confusing; for example, the model input and outcomes are not clearly described. Also, you may want to expand with more details on the method for the cohort simulation using the fitted Markov model.

**Response:**

We thank the reviewer for this insightful feedback. We have thoroughly revised the 'Model Strategy' and 'Model Parameters' sections to enhance the clarity, structure, and completeness of the model description. The revisions were made directly to the original text to improve the narrative flow. Key improvements include:

1. Reorganizing the description into logical subsections for better clarity: " The Markov Model and Cohort Simulation ", "Model Input Parameters", and "Model Calibration, Validation, and Outcomes".

2. Providing a structured overview of all model input parameters and their sources.

3. Clearly defining the model's primary outcomes (Costs, QALYs, ICURs).

#### Response to Reviewer Comment #4

**Comment:** “Page 16. 'By iteratively adjusting the symptom probability in the model, we ensured that the distribution of cases presented at each stage aligned closely with the reported distribution of incident cases.' These words are confusing as well. What does it mean to iteratively adjust the symptom, and what are the iterations during the calculation?”

**Response**:

We thank the reviewer for this valuable question. We apologize for the lack of clarity in our original description and are pleased to provide the following clarification.

The process described is a formal model calibration. The term "symptom probability" refers to a key natural history parameter in our model: the annual likelihood that a woman with undiagnosed breast cancer at a specific stage will develop symptoms leading to clinical diagnosis.(Table.4)

The **iterative adjustment** process was conducted as follows:

1. We ran the no-screening scenario in the model.
2. We compared the age-specific breast cancer incidence rates predicted by the model against the real-world age-specific incidence rates from Table 2 (Wang et al., 2022).
3. If the model's output did not match the target data well, we adjusted the symptom probabilities. For example, if the model predicted too few late-stage cancers, we decreased the symptom probability for early stages. This allowed more simulated cancers to progress to later stages before clinical diagnosis. (Sun, Legood, Sadique, Dos-Santos-Silva, & Yang, 2018)
4. We repeated this process until the model's output closely aligned with the target distribution. This required 42 iterations, achieving a satisfactory goodness-of-fit (X^2^ = 2.366,p-value > 0.05).

The final set of parameters from this calibration ensures our model's "no-screening" baseline accurately reflects real-world disease progression.

#### Response to Reviewer Comment #5

**Comment:** Page 16. Although it is mentioned in the paper, “A goodness-of-fit test was conducted to evaluate the similarity between the two curves.” But there are no hypothesis testing results or conclusions stated in the paper, i.e., reject or fail to reject the null hypothesis, test statistic, and p-value. The paper only mentioned, “This validation approach helps,” but no words mentioning whether the validation is successful or not.

**Response**:

We sincerely thank the reviewer for this astute observation and constructive critique. We agree that the initial description of the goodness-of-fit test was incomplete, as it lacked the specific statistical results necessary to conclusively demonstrate the model's validity. We apologize for this oversight.

In response to this comment, we have thoroughly revised the manuscript to provide a clear and quantitative assessment of the model's fit. The changes are as follows:

we have replaced the vague statement with a concise summary of the hypothesis test.

The goodness-of-fit between the model's final output and the observed data was formally assessed using a Chi-square goodness-of-fit test. The test, conducted across 10 age strata (9 degrees of freedom), yielded a test statistic of χ² = 2.366 with a p-value < 0.05. This non-significant result indicates no statistically significant difference between the model-predicted and the observed age-specific incidence distributions, supporting the model's external validity."

We have significantly expanded the Supplementary Appendix A.4: "Model Calibration and Validation" to provide a comprehensive account of the entire process. This new section includes:

1. A detailed, step-by-step description of the iterative calibration methodology.
2. The full results of the Chi-square goodness-of-fit test, and a calibration plot (Supplementary Figure 1) visually comparing the model-predicted incidence curve against the empirical data from the China National Cancer Center.

The reviewer is kindly referred to Supplementary Appendix A.4 for a complete and transparent presentation of the calibration targets, process, and the rigorous validation that underpins our model's natural history module.

We believe these revisions have fully addressed the reviewer's concern by providing the necessary hypothesis testing results and a definitive conclusion regarding the model's successful validation. We are grateful for this comment, which has undoubtedly strengthened the clarity and rigor of our manuscript.

#### Response to Reviewer Comment #6

**Comment:** Page 17.The title of Table 3 is “Average annual progression probability of breast cancer staging”, which needs to be adjusted since the table also includes other information beyond transition probabilities. Besides,“RR of invasive cancer from DICS” has a value of 2.02, which is, of course, not a probability, and there is no interpre- tation of the number,oraninterpretation of what is RR’smeaningisinthemain text (Is it relative risk?If so, “RR” needs to be stated in full when you first time usingit).Thedescriptionhereneedstobefine-tuned,andthetabletitleneedsto be adjusted.

**Response:**

We sincerely thank the reviewer for this insightful comment regarding the title and content of Table 3. We agree that the original title, “Average annual progression probability of breast cancer staging,” was not fully representative of the table’s content, which includes not only transition probabilities but also other parameters such as post-treatment mortality and a relative risk value.

In response, we have revised the table title to:

“Key Transition and Risk Parameters in the Markov Model for Breast Cancer Screening”

Additionally, we have clarified the term “RR” in both the table and the main text. “RR” stands for Relative Risk, which in this context represents the relative risk of developing invasive cancer among women diagnosed with Ductal Carcinoma In Situ (DCIS) compared to those without DCIS. The value of 2.02 was derived from the study by (Wong, Kuntz, Cowling, Lam, & Leung, 2007), which was referenced in our model. We have now explicitly stated this interpretation in the table footnote and in the Methods section under Table3 to enhance clarity.

The updated table footnote now reads:

“RR: Relative Risk of progressing from DCIS to invasive breast cancer, as derived from (Wong et al., 2007) A value of 2.02 indicates that women with DCIS have a 2.02 times higher risk of developing invasive cancer compared to those without DCIS.”

We believe these revisions improve the accuracy and interpretability of the table and thank the reviewer again for the valuable feedback.

#### Response to Reviewer Comment #7

**Comment:**

Page 19. Table 5, the reference to “Shenzhen breast cancer screening program” should be corrected.

**Response:**

We appreciate the reviewer's feedback. The costs in Table 5 are primary data from the "Shenzhen Municipal Health Commission Breast Cancer Screening Program (2021–2023)"—the very program under evaluation. They represent actual costs from the program and are not literature-based estimates. The source has been revised to explicitly indicate this direct origin.

#### Response to Reviewer Comment #8

**Comment:**

Page 26. Figure 3, this is the key graph in this paper, but it is very difficult to recognize the related screening strategy from the plot. Additionally, the phrases “upper-left region” and “lower-right corner” don’t match what the figure actually shows—no points appear in either quadrant. A better summary and description of the plot is needed.

**Response:**

We sincerely thank the reviewer for this insightful and constructive feedback regarding Figure 3, which is indeed central to our cost-utility analysis. We acknowledge that the original presentation and description could be significantly improved for clarity and accuracy.

In response, we have taken the following actions:

We have completely regenerated the cost-effectiveness scatter plot (now presented below for your review). Direct labeling of the five undominated strategies on the plot itself, eliminating the need for cross-referencing with a separate table or legend. And We have thoroughly revised the description in the "Cost-Utility Analysis" section (Results) to accurately reflect the visual data and provide a more precise interpretation. The phrases "upper-left region" and "lower-right corner" have been replaced with a more technically accurate description.

The revised text now reads:

Figure 3 presents the cost-utility scatter plot of the 27 simulated screening strategies. The vertical axis represents the total cost per person, and the horizontal axis represents the effectiveness in Quality-Adjusted Life Years (QALYs) per person. Strategies that are less effective and more costly than a combination of other strategies (i.e., dominated strategies) are plotted primarily in the top-left area of the graph relative to the cost-effectiveness frontier. The cost-effectiveness frontier, formed by the undominated strategies, runs from the bottom-left to the top-right. The undominated strategies are explicitly labeled in the figure and are, in order of increasing effectiveness: (CBE+BUS)+MAM/3year/45_65, (CBE+BUS)+MAM/1year/40_65, (CBE+BUS)+MAM/1year/35_65, (CBE+BUS)+MAM/1year/35_69, and (CBE+BUS)+MAM/1year/35_74. Their respective Incremental Cost-Utility Ratios (ICURs) are presented in the text.

We believe these revisions have substantially improved the clarity and interpretability of this key figure and its accompanying narrative. Thank you again for prompting these essential improvements.

#### Response to Reviewer Comment #9

**Comment:** Pages 30 and 32. Figure 8 is displayed in the main text, but is not mentioned in any text. On page 30, you may want to describe what the color (red VS. green) means, one or two red dots are further apart from the confidence interval bound ellipse and the associated possible interpretation, etc.

**Response:**

We sincerely thank the reviewer for this excellent observation. We apologize for the oversight in not previously describing Figure 8 in the main text. In response to this comment, we have now integrated a detailed description of the figure into the 'Sensitivity Analysis' section on page 30.

The added text reads:

"Figure 8 presents the incremental cost-utilities scatter plot from the probabilistic sensitivity analysis comparing the CBE+BUS_MAM/2year/35_65 strategy versus the CBE+BUS_MAM/3year/45_65 strategy. Each point represents the result of one Monte Carlo iteration, with green points indicating iterations where the ICUR falls below the WTP threshold (537,000 CNY/QALY), and red points indicating iterations where the ICUR exceeds the threshold. The 95% confidence ellipse illustrates the joint uncertainty around the mean incremental cost and effectiveness. The plot visually confirms that in 76% of the iterations (green points), the CBE+BUS_MAM/2year/35_65 strategy is cost-effective compared to the alternative. The presence of a few red dots distant from the main confidence ellipse reflects the tail-end of the parameter distributions in our model (e.g., combinations of high costs and low utilities), which is an expected outcome of the probabilistic analysis and represents the full range of uncertainty."

#### Response to Reviewer Comment #10

**Comment:**

Page 35. “with an ICUR of 140,915 CNY/QALY-well below China’s societal WTP threshold of 537,000 CNY/QALY. ” Based on your previous statement, 53,7000 CNY is calculated from the Shenzhen per capital GDP value, not the whole of Chinese societal WTP threshold.

**Response:**

We sincerely thank the reviewer for this meticulous correction. The reviewer is absolutely right. The Willingness-to-Pay (WTP) threshold of 537,000 CNY is indeed derived from three times the Shenzhen-specific per capita GDP, and not a national threshold for China.

We have now corrected this inaccurate statement in the manuscript

#### Response to Reviewer Comment #11

**Comment:**

The conclusions from comparisons between CBE+BUS MAM/2year/35-65 with no screen and CBE+BUS MAM/3year/45-65 with no screen are less interesting, especially in Shenzhen city with relatively higher GDP and hence higher threshold for cost-effectiveness.

**Response:**

We sincerely thank the reviewer for this insightful comment, which rightly points out that in a high-GDP city like Shenzhen, the value of screening versus no screening is often assumed. We agree that the more pressing and policy-relevant question in such settings is to identify the most efficient screening strategy among various alternatives.

In response to this valuable feedback, we would like to clarify the intent and structure of our analysis:

Although Shenzhen's screening program has been in operation, a formal cost-effectiveness evaluation has been absent. Our study fills this critical gap by providing robust evidence that the program constitutes a sound investment, thereby justifying the continued allocation of public health resources.

More importantly, our incremental analysis among the 27 strategies led to the identification of the optimal strategy: the current Shenzhen program (CBE+BUS_MAM/2year/35-65) is not merely cost-effective, but represents a strategically optimal choice. When compared directly against a plausible, less intensive alternative (CBE+BUS_MAM/3year/45-65), the current strategy yields an incremental cost-utility ratio (ICUR) of 518,121 CNY/QALY, which remains below the willingness-to-pay (WTP) threshold. In contrast, other more intensive strategies (e.g., those involving annual screening) were found not to be cost-effective.

Therefore, our study provides robust evidence to support the sustainability and specific design of Shenzhen's existing screening program. It demonstrates that the decision to start at age 35 and screen biennially represents a cost-effective balance between early detection and resource efficiency.

In light of the reviewer's comment, we have revised the Discussion and Conclusion section to more clearly frame our study's contribution around this theme of optimizing an established screening program, ensuring our conclusions are both novel and directly actionable for policy.

#### Response to Reviewer Comment #12

**Comment:**

Numerous citation and bibliography errors remain. Only two examples are given here,

1. In-text citations (page 15) - "Wong et al.'s research" lacks the publication year; the citations "

2. (Wu et al., 2017)” and "(Y Huang et al., 2013)” are repeated in the same sentence and should appear only once.

Reference list – Several entries do not follow the required style:

1. Page 42, "wuli, chenguozhen,..."- authors' surnames need initial capitals ("Wuli, Chenguozhen, ...”).

2. Some records omit journal titles or volume/issue details. i.e., "Zulfiqar, M., Rohazly, I., Rahmah, M. J. B. i., & journal, i. (2011). Do the majority of Malaysian women have dense breasts on mammogram? 7(2)."

**Response:**

We thank the reviewer for highlighting these formatting inconsistencies. We have thoroughly addressed all the points raised:

1.We have ensured that all narrative citations (e.g., "Wong et al.'s research") are now followed by the publication year in parentheses. And we have consolidated all repeated citations within a single sentence, as per the journal's style guide.

2.We have performed a comprehensive check of the entire reference list. This included ensuring correct capitalization of all author names and completing missing journal titles and volume/issue details for all entries, including the examples provided.

We have carefully reviewed and formatted the entire manuscript and bibliography to ensure full compliance with the journal's style guide. We apologize for these oversights.

Sun, L., Legood, R., Sadique, Z., Dos-Santos-Silva, I., & Yang, L. (2018). Cost-effectiveness of risk-based breast cancer screening programme, China. *Bull World Health Organ, 96*(8), 568-577. doi:10.2471/blt.18.207944

Wang, J., Greuter, M. J. W., Zheng, S., van Veldhuizen, D. W. A., Vermeulen, K. M., Wang, Y., . . . de Bock, G. H. (2022). Assessment of the Benefits and Cost-Effectiveness of Population-Based Breast Cancer Screening in Urban China: A Model-Based Analysis. *Int J Health Policy Manage, 11*(9), 1658-1667. doi:10.34172/ijhpm.2021.62

Wong, I. O., Kuntz, K. M., Cowling, B. J., Lam, C. L., & Leung, G. M. (2007). Cost effectiveness of mammography screening for Chinese women. *Cancer, 110*(4), 885-895. doi:10.1002/cncr.22848
