# Supplementary material for: A cost-effective breast cancer screening strategy for Urban China: Findings from a Shenzhen-based modeling study
Source: PLoS One. 2026 Mar 12;21(3):e0344253. doi: 10.1371/journal.pone.0344253 (PMC12981465; doi:10.1371/journal.pone.0344253)
Supplement: Attachment — Submitted filename: Response to the Academic Editor.docx [file pone.0344253.s013.docx]

#### Response to Comments

We thank the Academic Editor for the constructive feedback, which has helped to clarify key methodological aspects of our study. Below are our point-by-point responses to the specific editorial comments.

#### Response to Academic Editor Comments #1

**Comment :**

Supplementary Figure 1 was not uploaded.
**Response:**

Thank you for catching this oversight. Supplementary Figure 1 (Model Calibration Fit Plot) has now been uploaded with the revised submission files.

#### Response to Academic Editor Comments #2

**Comment :**

Notation in Table 8 – “CBE + (US-MAM)”.

**Response:**

We have reviewed and corrected the notation. In the revised Table 8, this strategy is now labeled as (CBE + BUS) + MAM.

#### Response to Academic Editor Comments #3

**Comment 3:**

Clarification on “age-specific incidence” vs. homogeneous transition probabilities.

**Response:**

We appreciate this important point, which aligns with Reviewer #1’s query (Comment #3). We have substantially revised the relevant sections to eliminate confusion.

- **In Supplementary Appendix A.2.2,** we now explicitly label the transition matrix as **P(a)**, where ‘a’ denotes the cohort’s attained age. We have structured the description under the matrix into four clear components:
  1. **Time-homogeneous Elements:** Constant annual progression probabilities between clinical cancer stages (e.g., Stage I→IV), directly sourced from Table 3.
  2. **Self-Transition Elements:** Diagonal probabilities calculated as complements of outgoing transitions.
  3. **Age-dependent Composite Elements:** Transitions from Healthy/DCIS states, calculated in a two-step process using age-specific mortality (Table 1), incidence (Table 2), diagnostic pathway (Table 8), and stage distribution (Table 4).
  4. **Mortality Transitions:** Sum of age-specific non-breast cancer mortality (Table 1) and stage-specific breast cancer mortality (Table 3).
- **In the Discussion section (Limitations subsection),** we have added a new paragraph (see below) to transparently address this modeling choice, justify it based on calibration performance, and acknowledge it as a standard simplification in the absence of longitudinal, age-stratified progression data.

***New text for Limitations：***
Fourthly, our model assumes time-homogeneous transition probabilities between clinical stages, following the framework of Wong et al.[21]. While incidence and mortality are age-dependent, internal progression rates are constant. This may not fully capture variations in biological aggressiveness across age groups, a simplification necessitated by the scarcity of age-stratified progression data for Chinese women. Future studies with longitudinal data are needed to refine these probabilities.
